# Impact of the 7/14/2016 Nice terrorist attack on pediatric emergency department visits thanks to syndromic surveillance: a descriptive study

**DOI:** 10.3389/fpubh.2023.1248993

**Published:** 2023-10-17

**Authors:** Arnaud Fernandez, Laure Meurice, Florian Franke, Cecile Vuillermoz, Morgane Gindt, Florence Askenazy, Stéphanie Vandentorren

**Affiliations:** ^1^University Department of Child and Adolescent Psychiatry, Children’s Hospitals of Nice CHU-Lenval, Nice, France; ^2^CoBTek, Université Côte d'Azur, Nice, France; ^3^Santé publique France, French National Public Health Agency, Nouvelle-Aquitaine Regional Office, Bordeaux, France; ^4^Santé Publique France, French National Public Health Agency, Regional Unit (CIRE, Provence-Alpes-Côte d'Azur and Corsica), Marseille, France; ^5^Sorbonne Université, INSERM, Institut Pierre Louis d’Epidémiologie et de Santé Publique (IPLESP), Social Epidemiology Research Team, Paris, France; ^6^Université Bordeaux, Inserm, UMR1219, Vintage Team, Bordeaux, France; ^7^Santé publique France, French National Public Health Agency, Saint-Maurice, France

**Keywords:** stress, child and adolescent psychiatry, psycho-trauma, syndromic surveillance, terrorist attack

## Abstract

**Objective:**

Study the impact of 14th July 2016 Nice terrorist attack on Pediatric Emergency Department (PED) visits by youth under 18 years of age.

**Methods:**

PED visits diagnoses (ICD10) were clustered and analyzed based on retrospective data from the syndromic surveillance system of the Children’s university hospital of Nice (Southern France). The studied period ranges from 2013 to 2019, i.e., 3 years before and after the terrorist attack of 14th July 2016.

**Results:**

Among 416,191 PED visits, the number of visits for stress in 4–17 years old appeared to increase in the 3 years after the attack compared to the 3 years before, particularly in September 2016 (acute effect) with 11 visits compared to an average of 2.3 visits per month from September 2013 to 2016 (*p* = 0.001827). In September 2017, we noticed 21 visits compared to an average of 4.8 visits per month during the following period (2013–2019). In 2017, PED visits for stress among 4–17 year olds were higher in comparison to the other years of the study: 107 visits compared to an annual average of 57.

**Conclusion:**

To our knowledge, this is the first study of the use of the pediatric care system before and after a terrorist attack involving syndromic surveillance. This suggests acute and long-term effects of the terrorist attack on PED use by youth for mental health issues. Further studies of the pediatric care system involving syndromic surveillance are needed in the context of mass violent events, such as terrorist attacks.

## Introduction

The city of Nice (south of France) was hit by an Islamist terrorist attack with a truck on French national day, on 14 July 2016. The death toll rose to 86 killed people, including 15 children with 458 injured and more than 30,000 witnesses of the event. According to international classifications (ICD 11 and DSM 5-TR), a terrorist attack is a grade event resulting in a potential psycho-trauma: a psycho-trauma exposure according to the DSM. Psycho-trauma, can have consequences on physical health, social relationships, and quality of life. They can also generate psychiatric disorders. Post-traumatic Stress Disorder (PTSD: DSM 5-TR, 2023) is the most studied of them. Other disorders can occur and be associated with PTSD, such as anxiety, mood disorders like depression, addiction and Attention Deficit Disorder (ADD), with or without Hyperactivity which can all occur past the initial traumatic event and can vary long after (1–3). Psychological and psychiatric consequences of a sole exposure to a traumatic event, as terrifying and shocking as it could be [type 1 trauma according to Terr (4)], can be extremely severe with children, with a possible toll on their development (5). These consequences can differ from an adult population and can vary according to the child’s age and depend on his entourage’s reactions, most notably his parents’ (6).

In Nice, the psychiatric impact after the terrorist attack of 14th July 2016 was heavy within the pediatric population. An epidemiologic clinical study led by the Nice University service of the child and adolescent psychiatry, showed that 62% of the 271 children admitted suffered from PTSD, regardless of their age (7, 8). This terrorist attack happened not far (around 200 m) from the Children’s university hospital of Lenval. It includes pediatric emergencies, located on one single site and which has admitted on average around 60,000 pediatric emergencies per year since 2013 (fourth children’s admittance in France). During and after the attack, the Lenval Hospital had to produce an exceptional and swift response to the gravity of the event (several fatal casualties) and the admittance high demands (9–11).

Children are indeed an extremely vulnerable population, this coming from their lower emotional maturity, which does not allow them to comprehend the event they have experienced and does not allow them to make sense of it. Moreover, exposure to traumatic events in childhood is a risk factor that increases the chances of developing PTSD later in life (12). The emotional state of the adult accompanying the child is also an important factor, regulating the possible apparition of disorders in the wake of a psycho-trauma. In the context of a terrorist or mass violence attacks, taking this into consideration is of utter importance as parents are often affected by the same event. On a pathopsychological level, the different zones of the child’s brain show an unequal growth (13). The frontal zones, which show a subsequent growth (until the end of the adolescence), can be more sensitive to later psycho-traumas, opposed to the hippocampal regions, which are generally more affected by stress induced events from early childhood (14, 15).

In the wake of the event (from the second day to the first month after the psycho-trauma), the child can develop an acute stress, anxiety, depression, regressive behaviors, and somatizations. After a month, the child can experience nightmares, somatizations, disturbed cognitive schemes with a refusal to learn, fear of separation and depression, delayed growth, or regressive behaviors (16). Before the age of 3, the child usually reacts displaying biophysiological, motor, eating and attachment (development, anorexia, insomnia, fits of rage and cries or stillness) disorders. After the age of 3, we can observe intrusive reminiscences of the psycho-trauma, behaviors featuring avoidance of fear of separation (possibly leading to an anxious school refusal), or aggressiveness and regressiveness. However, these consequences can be altered with an adapted and early care of the child (17, 18).

The link between somatic symptoms and post-traumatic stress has been studied in an adult population (19). Exposure to traumatic events in childhood has serious and damaging effects on the mental and physical health of adults (20). Similar adverse effects probably exist in children, but data are lacking. Using a syndromic surveillance system should allow for better measurements (and better care) for patients affected by these type of events (21). Yet, no studies of this kind is currently available to our knowledge to measure the impact of psycho-trauma on children and its consequences on the use of the pediatric care system. Terrorist attacks have been more frequent these last decades worldwide (even among pacified countries). Hence, understanding their impact could be useful to the French health care system but also to European and worldwide countries.

The aim of this study is to investigate the psycho-traumatic impact of the Nice terrorist attack on children under the age of 18 by exploring changes in complaints from users of the Nice (France) pediatric healthcare system (pediatric emergency departments) after this mass violence event.

## Materials and methods

### Study scheme

This study is retrospectively based on the data from the pediatric emergencies from the Children’s university hospital of Lenval, from 2013 to 2019, meaning 3 years before and after the terrorist attack of 14 July 2016.

### Setting

Nice is France’s 5th largest city in terms of population. The French National Institute for Statistics and Economic Studies (Insee) estimated that in 2016, Nice had 54.667 children under the age of 15 (15.8% of the total population of 345,998). Nevertheless, the Lenval hospital is the only establishment in the city to deal with pediatric emergencies.

### Collecting data

Collecting data came from the e-SurSaUD^®^ database, which compiles emergency room visits (“résumés de passages aux urgencies”—RPU) of the Children’s University Hospital of Lenval. Each day, every emergency room visits are automatically sent to “Santé Publique France” (the French National Public Health Agency) as part of the syndromic surveillance system.^1^ The main variables coming from the emergency room visits are administrative variables (identification number of the hospital, date, and time of admittance, etc.), demographic variables (gender, date of birth, etc.) and medical variables (main diagnosis, associated diagnosis, etc.). Medical diagnoses are coded according to the 10th revision of the International Classification of Diseases (ICD10) and syndromic clusters, which include one or several codes, are built by the French National Public Health Agency for health monitoring and epidemiology surveillance. Collected variables for the study include: the patient’s age (at the time of consultation), the date of emergency room admittance and the main and/or associated diagnosis following the patient’s admittance (several diagnoses are possible). With this device, we have collected data for the city of Nice.

### Analyzing data

The analysis was focused on the emergency room visits of the Lenval university hospital for 26 syndromic clusters, comprised of diagnoses given at the pediatrics emergencies, each including CIM codes between 1 and 2,487 (see Supplementary Appendix 1). Out of those 26 specific syndromic clusters, 10 are dedicated to patients aged between 0- and 3-year-olds (Supplementary Appendix 1). We have also considered the “any causes visits” criteria, which includes all coded visits regardless of the diagnosis.

To study the event’s acute effects (the differences seen between a time of 3 months before and after the attack) and the delayed ones (the differences seen between a time of 3 years before and after the attack), we have considered the time evolution of the number of visits and the proportion of activity for each of the syndromic clusters on a weekly and monthly basis. We used *Chi2* and Wilcoxon tests to assess a comparison of the proportion of emergency department visits based on before and after the event. The patients’ age varied between 0- and 3-year-olds and 4- and 17-year-olds. The 0–3-year-old group included newborns, infants, and non-verbal children with a different symptomatic expression from older children and represented a major proportion of emergency department visits (53.5% of the total number of 0–17-year-old visits during the same period). On the other hand, the 4–17-year-old group was not analyzed in homogeneous subcategories to avoid any risks of over-accentuating the representation of this age group, which only represented 46.5% of the total number of 0–17-year-old visits.

## Results

### Global activity

On this period, 416,191 visits were registered at the Lenval Hospital (222,748 visits from the 0- to 3-year-olds and 193,443 visits from the 4- to 17-year-olds), giving an average number of 59,456 visits per year (31,821 for the 0- to 3-year-olds and 27,635 for the 4- to 17-year-olds, see Supplementary Appendix 2).

### Acute effects

Analyzing the syndromic clusters of the 0–3 years-old and the 4–17 years-old over the period of 3 months after the 14 July 2016 attack did not highlight any acute effects. Analyzing the acute effects (3 months post-attack) considering a one-month period, we find a significant difference for the month of September 2016 (compared to September 2013, 2014, and 2015) for the variable stress among 4–17 year-olds. For the months of September 2013 to 2015 we find an average of 0.08 passages per day versus 0.37 per day in 2016 (*p* = 0.001827).

### Delayed effects

**Figure 1:**
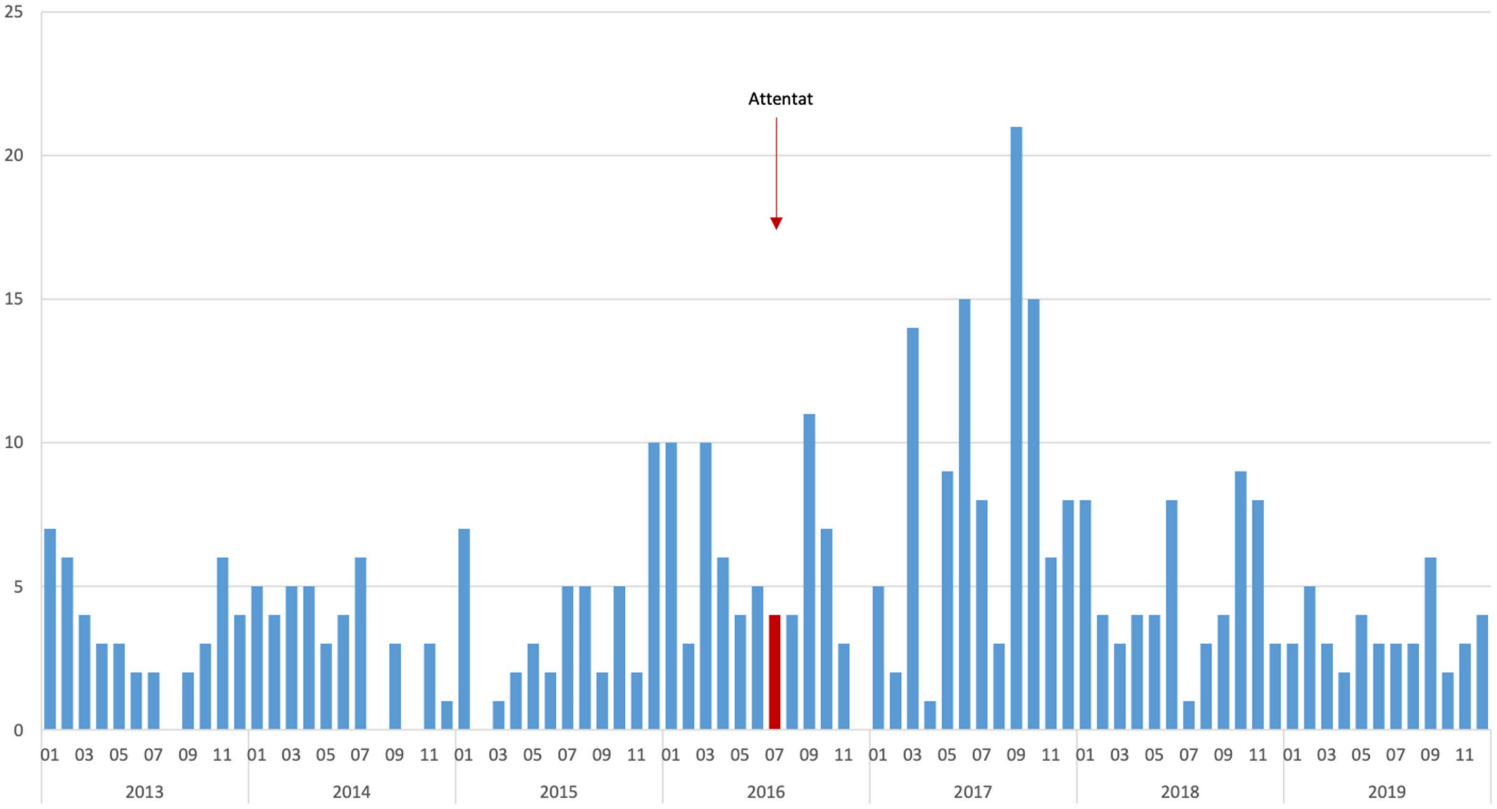
Monthly number of emergency department visits for stress in the 4–17-year-old children, Lenval university hospital, 2013–2019.

Within the 0–3-year-old group, we could not observe any delayed effects during the 3-year period following the attack as opposed to the 3-year period prior to the event. This very same analysis of the 3-year period following the attack allowed us to measure a spike of emergency department visits for stress in September 2017 within the 4–17-year-olds (Figure 1). This matches the time children were back to school after the one-year anniversary of the attack. Twenty one visits for an average per month over the period of study of 4.8 visits, a number largely outside the 95% confidence interval normally observed for this monthly average (CI: [4.0–5.5]). This meant a proportion of 0.9% visits for the 4–17-year-olds (against an average 0.2% over the 2013–2019 period; see Supplementary Appendix 2). However, no similar spike for emergency department visits for stress within the 4–17-year-olds was registered for the months of September in 2018 (4 visits) and 2019 (6 visits). More generally, in 2017, there were far more emergency department visits for stress within the 4–17-year-olds in comparison to the other years of the study: 107 visits for an annual average of 57. The details of the 17 ICD-10 codes constituting the stress syndromic cluster are presented in Supplementary Appendix 3.

## Discussion

No differences in the number of visits for the 26 syndromic clusters was shown between the 3-month period prior to and the 3-month period following the attack analyzing the 3 months as a grouped variable. Considering a one-month period, we found a significant difference for the month of September 2016 (compared to September 2013, 2014, and 2015) for the variable stress among 4–17 year-olds (*p* = 0.001827). This period was chosen as it usually matches a time delay, after which the appearance of PTSD after a traumatic event is very unlikely [3.5% according to Santiago et al. (22)]. This means that an immediate effect would be measured after the attack on the sole basis of emergency department visits. The organization of a psychiatric care unit right after the attack, put up by the university’s child and adolescent psychiatry service of Nice in combination with the help of regional and national services provided to the affected population, seems to have been an appropriate response (23, 24). Desite this, the acute effect measured in terms of number of visits could have been greater. On one hand, data (number and patterns of the visits) from the public health emergency preparedness and response department [“Établissement de Préparation et de Réponse aux Situations Sanitaires Exceptionnelles (EPRUS)] and from medical and psychological emergencies crisis units [cellules d’urgence médico-psychologique (CUMP)] were not counted within pediatrics emergencies. On the other hand, a beneficial effect may have occurred during this time of limited emergency response, which could have reduced the necessity for systematic emergency department visits for stress right after this post-attack period.

During the 3 years that followed the attack, the number of emergency department visits for stress within the 4–17-year-olds seemed to rise in comparison to the 3 years prior to the attack, most notably in 2017 (one-year anniversary of the attack). We have also observed a spike of visits for stress within 4–17-year-olds in September 2017. This phenomenon means there was a probable rebound of anxiety following the first national commemoration of the event (25), which took place on 14 July 2017. Following this, going back to school for children probably meant an additional source of stress (as we have already assumed for the peak of stress among 4–17 year-olds at the start of the school year in September 2016, 2 months after the attack), combined with the stress experienced during the national commemoration, which had happened only 2 months before. This security obsessed context, caused by the post-attack security plan (“Vigipirate”) and its strict policy forbidding parents to accompany their children within the school premises, most probably had its effects in terms of stress. Indeed, Virginia Gil-Rivas et al. (26) have demonstrated that 1 year after the 9/11 attacks in 2001, the traumatic impact after the event was not limited to teenagers that were exposed to it but that it also had repercussions to teenagers that had psychiatric antecedents or experienced learning difficulties, while also taking into consideration their parents’ anxiety level. Thus, in our population that goes to school (from kindergarten to high school), going away on holiday to avoid the commemoration (avoidance syndrome) probably had a significative impact and meant an increase of emergency visits when those children came back from their holiday and went back to school after this summer of commemoration of the attack. This is confirmed as for the years that followed, 2018 and 2019, no such increase of visits for stress was registered as it was in 2017. Furthermore, we have double checked with SurSAUD’s data (for stress syndromic cluster regarding the 5–14-year-old category) coming from the PED of Marseille (a city that was not directly affected in this period by a terrorist resulting with a similar number of casualties) and from the national level (Whole French territory). We have not registered any increase of visits for stress over the whole year of 2017, nor for the month of September in particular. This specific argument validates the observed effects at the pediatric emergency department of Nice during the anniversary of the terrorist attack.

Thereby, analyzing data from the pediatrics emergencies allowed us, retrospectively, to issue a warning on the probable health risk, depending on age (stress within the 4–17-year-olds) and to expose the magnitude of its effect and its persistence in time (more than 1 year after the event).

### Limitations

Although this increase of pediatric emergencies visits for stress may suggest a mental health impact of the 14/07/2016 terrorist attack on the pediatric population of Nice, we cannot ascertain there is direct link of causality.

Indeed, at the time, a device for a live syndromic surveillance that would report all reasons for emergencies visits directly or indirectly linked to the terrorist attack was not set up right after the event nor for a longer period afterwards. We are also not able to analyze in a qualitative manner all the factors triggering or favoring these emergencies visits for stress. PTSD is the psychiatric disorder associated with the highest frequency of somatic complaints (27). Among young people, the most studied potentially traumatic mass events have been disasters triggered by natural hazards (Hurricane Katrina and the Japanese earthquakes). Somatic complaints described and associated with post-traumatic stress include sleep disorders, fatigue, headaches and abdominal pain (28–30). The link between stress and somatic complaints in the general pediatric population after a terrorist attack, however, has not yet been studied. Likewise, an under-estimation of these visits for stress may have intervened if, in a post-attack period and during the anniversary period, a somatic complaint was not directly attributed to its underlying stress (under-diagnosis and diagnosis with a delayed effect during an ulterior visit).

It is important to note in that sense that during this study period, there was no caring team specialized in child and adolescent psychiatry at the pediatric emergencies of the Lenval university hospital for children. Instead, there was a dedicated team of pediatrists with a child and adolescent psychiatry resident on duty and a senior practitioner on call from home (31). This context could also have led to under-diagnosis of stress syndromic cluster.

To conclude, to our knowledge, this is the first study of the use of the pediatric care system before and after a terrorist attack involving syndromic surveillance in a context of catastrophe with a mass trauma. Our results suggest acute and long-term effects of the terrorist attack on PED use by youth for mental health issues. A live syndromic surveillance device (21, 32) and an analysis of data from the field (private practice medicine, emergency medical service), associated with data from other sites in the country that were not affected, would allow us to understand better the links between the different variations of solicitations for emergency care at the hospital from the population and the effect of the studied catastrophe on the health of that population. A dedicated consultation center was set up by the child and adolescent psychiatry department of the university hospital after this event to measure the effects caused by this psycho-trauma and its long-term negative consequences (33). Further studies will be conducted to measure the benefit it may procure, both on an individual and more global level, for young people admitted for care.

## Data availability statement

The raw data supporting the conclusions of this article will be made available by the authors, without undue reservation.

## Ethics statement

Ethical approval was not required for the study involving humans in accordance with the local legislation and institutional requirements. Written informed consent to participate in this study was not required from the participants or the participants’ legal guardians/next of kin in accordance with the national legislation and the institutional requirements.

## Author contributions

AF, FA, and SV contributed to conception and design of the study. LM and FF organized the database and performed the statistical analysis. AF wrote the first draft of the manuscript. AF, LM, FF, CV, MG, FA, and SV wrote sections of the manuscript. All authors contributed to manuscript revision, read, and approved the submitted version.

## Footnotes

^1^ https://www.santepubliquefrance.fr/surveillance-syndromique-sursaud-R
