# Supplementary material for: Impact of the 7/14/2016 Nice terrorist attack on pediatric emergency department visits thanks to syndromic surveillance: a descriptive study
Source: Front Public Health. 2023 Oct 17;11:1248993. doi: 10.3389/fpubh.2023.1248993 (PMC10616791; doi:10.3389/fpubh.2023.1248993)
Supplement: Supplementary file 1 [file Data_Sheet_1.docx]

| **AGES** | **SYNDROMIC CLUSTERS** | **ICD-10 number of codes** |
| --- | --- | --- |
| 0-17 | ASTHMA | 6 |
| 0-17 | BURNS | 120 |
| 0-17 | CONJONCTIVITIES | 24 |
| 0-17 | HEART FAILURE | 1 |
| 0-17 | DERMATOLOGICAL DISEASES (OTHER) | 8 |
| 0-17 | FEVER AND RASH | 11 |
| 0-17 | SUDDEN FEVER | 6 |
| 0-17 | GASTROENTERITIS | 8 |
| 0-17 | HYPOTENSION (LOW BLOOD PRESSURE)-SHOCK | 5 |
| 0-17 | DYSPNEA, RESPIRATOIRE FAILURE | 6 |
| 0-17 | FAINTNESS | 6 |
| 0-17 | NEUROLOGICAL DISORDERS (OTHER) | 51 |
| 0-17 | STRESS | 17 |
| 0-17 | TRAUMA | 2487 |
| 0-17 | ARRHYTHMIA OR CARDIAC CONDUCTION DISORDER | 5 |
| 0-17 | ANXIETY DISORDERS | 58 |
| 0-3 | DERMATOLOGICAL DISEASES | 17 |
| 0-3 | SUDDEN INFANT DEATH SYNDROME | 3 |
| 0-3 | EXCESSIVE INFANT CRYING | 1 |
| 0-3 | EATING DISORDERS | 11 |
| 0-3 | COMMUNICATION DISORDERS | 2 |
| 0-3 | BEHAVIORAL DISORDERS | 3 |
| 0-3 | MOTOR DISORDERS | 4 |
| 0-3 | BREATHING PROBLEMS | 5 |
| 0-3 | SLEEPING PROBLEMS | 9 |
| 0-3 | SPHINCTER FUNCTION DISORDERS | 8 |
|  |  |  |
| **Appendix 1: Syndromic clusters according to age. 26 syndromic clusters have been defined, of which 10 were dedicated to the 0- to 3-year-old group.**   \| **MONTHS** \| **TOTAL** \| **TOTAL 0-3** \| **TOTAL 4-17** \| **STRESS 4-17** \| **STRESS 4-17** \| \| --- \| --- \| --- \| --- \| --- \| --- \| \| 2013-01 \| 5226 \| 2776 \| 2450 \| 7 \| 0,3% \| \| 2013-02 \| 4537 \| 2569 \| 1968 \| 6 \| 0,3% \| \| 2013-03 \| 4599 \| 2410 \| 2189 \| 4 \| 0,2% \| \| 2013-04 \| 4094 \| 2146 \| 1948 \| 3 \| 0,2% \| \| 2013-05 \| 4630 \| 2268 \| 2362 \| 3 \| 0,1% \| \| 2013-06 \| 5075 \| 2533 \| 2541 \| 2 \| 0,1% \| \| 2013-07 \| 4420 \| 2401 \| 2019 \| 2 \| 0,1% \| \| 2013-08 \| 4066 \| 2254 \| 1812 \| 0 \| 0,0% \| \| 2013-09 \| 4400 \| 2442 \| 1958 \| 2 \| 0,1% \| \| 2013-10 \| 4613 \| 2637 \| 1976 \| 3 \| 0,2% \| \| 2013-11 \| 4578 \| 2417 \| 2161 \| 6 \| 0,3% \| \| 2013-12 \| 5517 \| 3510 \| 2007 \| 4 \| 0,2% \| \| 2014-01 \| 4907 \| 2863 \| 2044 \| 5 \| 0,2% \| \| 2014-02 \| 4669 \| 2610 \| 2059 \| 4 \| 0,2% \| \| 2014-03 \| 4768 \| 2435 \| 2333 \| 5 \| 0,2% \| \| 2014-04 \| 4786 \| 2553 \| 2233 \| 5 \| 0,2% \| \| 2014-05 \| 4699 \| 2348 \| 2351 \| 3 \| 0,1% \| \| 2014-06 \| 4872 \| 2568 \| 2304 \| 4 \| 0,2% \| \| 2014-07 \| 4190 \| 2338 \| 1852 \| 6 \| 0,3% \| \| 2014-08 \| 4278 \| 2316 \| 1962 \| 0 \| 0,0% \| \| 2014-09 \| 4617 \| 2354 \| 2263 \| 3 \| 0,1% \| \| 2014-10 \| 5033 \| 2793 \| 2240 \| 0 \| 0,0% \| \| 2014-11 \| 4718 \| 2550 \| 2168 \| 3 \| 0,1% \| \| 2014-12 \| 6004 \| 3775 \| 2229 \| 1 \| 0,0% \| \| 2015-01 \| 5357 \| 2900 \| 2457 \| 7 \| 0,3% \| \| 2015-02 \| 5031 \| 2761 \| 2270 \| 0 \| 0,0% \| \| 2015-03 \| 4782 \| 2436 \| 2346 \| 1 \| 0,0% \| \| 2015-04 \| 4962 \| 2504 \| 2458 \| 2 \| 0,1% \| \| 2015-05 \| 4795 \| 2429 \| 2366 \| 3 \| 0,1% \| \| 2015-06 \| 4911 \| 2406 \| 2505 \| 2 \| 0,1% \| \| 2015-07 \| 4818 \| 2695 \| 2123 \| 5 \| 0,2% \| \| 2015-08 \| 4462 \| 2426 \| 2036 \| 5 \| 0,2% \| \| 2015-09 \| 4765 \| 2467 \| 2298 \| 2 \| 0,1% \| \| 2015-10 \| 4909 \| 2737 \| 2172 \| 5 \| 0,2% \| \| 2015-11 \| 5023 \| 2701 \| 2322 \| 2 \| 0,1% \| \| 2015-12 \| 5857 \| 3569 \| 2288 \| 10 \| 0,4% \| \| 2016-01 \| 5440 \| 2742 \| 2698 \| 10 \| 0,4% \| \| 2016-02 \| 4952 \| 2735 \| 2217 \| 3 \| 0,1% \| \| 2016-03 \| 5681 \| 2884 \| 2797 \| 10 \| 0,4% \| \| 2016-04 \| 4371 \| 2223 \| 2148 \| 6 \| 0,3% \| \| 2016-05 \| 4986 \| 2430 \| 2556 \| 4 \| 0,2% \| \| 2016-06 \| 4660 \| 2388 \| 2272 \| 5 \| 0,2% \| \| 2016-07 \| 4325 \| 2433 \| 1892 \| 4 \| 0,2% \| \| 2016-08 \| 4065 \| 2103 \| 1962 \| 4 \| 0,2% \| \| 2016-09 \| 4476 \| 2260 \| 2216 \| 11 \| 0,5% \| \| 2016-10 \| 4875 \| 2801 \| 2074 \| 7 \| 0,3% \| \| 2016-11 \| 5233 \| 2893 \| 2340 \| 3 \| 0,1% \| \| 2016-12 \| 5856 \| 3657 \| 2199 \| 0 \| 0,0% \| \| 2017-01 \| 5587 \| 2900 \| 2687 \| 5 \| 0,2% \| \| 2017-02 \| 4411 \| 2526 \| 1885 \| 2 \| 0,1% \| \| 2017-03 \| 5447 \| 2650 \| 2797 \| 14 \| 0,5% \| \| 2017-04 \| 4856 \| 2522 \| 2334 \| 1 \| 0,0% \| \| 2017-05 \| 5380 \| 2647 \| 2733 \| 9 \| 0,3% \| \| 2017-06 \| 5265 \| 2660 \| 2605 \| 15 \| 0,6% \| \| 2017-07 \| 4906 \| 2644 \| 2262 \| 8 \| 0,4% \| \| 2017-08 \| 4509 \| 2419 \| 2090 \| 3 \| 0,1% \| \| 2017-09 \| 4903 \| 2658 \| 2245 \| 21 \| 0,9% \| \| 2017-10 \| 5424 \| 3026 \| 2398 \| 15 \| 0,6% \| \| 2017-11 \| 4902 \| 2575 \| 2327 \| 6 \| 0,3% \| \| 2017-12 \| 6839 \| 4330 \| 2509 \| 8 \| 0,3% \| \| 2018-01 \| 5476 \| 3072 \| 2404 \| 8 \| 0,3% \| \| 2018-02 \| 5023 \| 2655 \| 2368 \| 4 \| 0,2% \| \| 2018-03 \| 4516 \| 2331 \| 2185 \| 3 \| 0,1% \| \| 2018-04 \| 4832 \| 2395 \| 2437 \| 4 \| 0,2% \| \| 2018-05 \| 4735 \| 2300 \| 2435 \| 4 \| 0,2% \| \| 2018-06 \| 4976 \| 2443 \| 2533 \| 8 \| 0,3% \| \| 2018-07 \| 4695 \| 2465 \| 2230 \| 1 \| 0,0% \| \| 2018-08 \| 4699 \| 2404 \| 2295 \| 3 \| 0,1% \| \| 2018-09 \| 5018 \| 2564 \| 2454 \| 4 \| 0,2% \| \| 2018-10 \| 5111 \| 2671 \| 2440 \| 9 \| 0,4% \| \| 2018-11 \| 5149 \| 2777 \| 2372 \| 8 \| 0,3% \| \| 2018-12 \| 6199 \| 3803 \| 2396 \| 3 \| 0,1% \| \| 2019-01 \| 5812 \| 3054 \| 2758 \| 3 \| 0,1% \| \| 2019-02 \| 5013 \| 2759 \| 2254 \| 5 \| 0,2% \| \| 2019-03 \| 5496 \| 2621 \| 2875 \| 3 \| 0,1% \| \| 2019-04 \| 4629 \| 2313 \| 2316 \| 2 \| 0,1% \| \| 2019-05 \| 5175 \| 2306 \| 2869 \| 4 \| 0,1% \| \| 2019-06 \| 5024 \| 2494 \| 2530 \| 3 \| 0,1% \| \| 2019-07 \| 4895 \| 2637 \| 2258 \| 3 \| 0,1% \| \| 2019-08 \| 4512 \| 2359 \| 2153 \| 3 \| 0,1% \| \| 2019-09 \| 4940 \| 2496 \| 2444 \| 6 \| 0,2% \| \| 2019-10 \| 5063 \| 2708 \| 2355 \| 2 \| 0,1% \| \| 2019-11 \| 5068 \| 2606 \| 2462 \| 3 \| 0,1% \| \| 2019-12 \| 5818 \| 3511 \| 2307 \| 4 \| 0,2% \|   **Appendix 2: Number of pediatric emergency department visits per month, before and after the terrorist attack (month highlighted in red) for the total number of young people, for the 2 age groups analyzed (0-3 and 4-17) and for the 4-17 stress cluster.** | | |
|  |  |  |
| **AGES** | **ICD-10 CODES** | **ICD-10 CODES** |
| 0-17 | Reaction to a severe stress factor, and adjustment disorders | F43 |
| 0-17 | Acute reaction to a stress factor | F430 |
| 0-17 | Light acute reaction to a stress factor | F4300 |
| 0-17 | Réaction aigüe moyenne à un facteur de stress | F4301 |
| 0-17 | Severe acute reaction to a stress factor | F4302 |
| 0-17 | Post-traumatic stress disorder | F431 |
| 0-17 | Adjustment disorders | F432 |
| 0-17 | Short depressive reaction | F4320 |
| 0-17 | Extended depressive reaction | F4321 |
| 0-17 | Mixed, anxious and depressive reaction | F4322 |
| 0-17 | Adjustment disorders with a predominance of other type of emotional disturbance | F4323 |
| 0-17 | Adjustment disorders with a predominance of behavioral disturbance | F4324 |
| 0-17 | Adjustment disorders with a combined emotional and behavioral disturbances | F4325 |
| 0-17 | Adjustment disorders with a predominance of other type of identified syndromes | F4328 |
| 0-17 | Other type of reactions to a severe stress factor | F438 |
| 0-17 | Reaction to a severe stress factor, with any specific mention | F439 |
| 0-17 | State of emotional shock, with any specific mention | R457 |

**Appendix 3: ICD-10 codes constituting the STRESS syndromic cluster**
